# Detecting the genetic link between Alzheimer's disease and obesity using bioinformatics analysis of GWAS data

**DOI:** 10.18632/oncotarget.19115

**Published:** 2017-07-08

**Authors:** Qi-Shuai Zhuang, Hao Zheng, Xiao-Dan Gu, Liang Shen, Hong-Fang Ji

**Affiliations:** ^1^ Shandong Provincial Research Center for Bioinformatic Engineering and Technique, School of Life Sciences, Shandong University of Technology, Zibo, P. R. China

**Keywords:** Alzheimer’s disease, obesity, bioinformatics, genome-wide association studies, single nucleotide polymorphisms, Gerotarget

## Abstract

Alzheimer's disease (AD) represents the major form of dementia in the elderly. In recent years, accumulating evidence indicate that obesity may act as a risk factor for AD, while the genetic link between the two conditions remains unclear. This bioinformatics analysis aimed to detect the genetic link between AD and obesity on single nucleotide polymorphisms (SNPs), gene, and pathway levels based on genome-wide association studies data. A total of 31 SNPs were found to be shared by AD and obesity, which were linked to 7 genes. These genes included PSMC3, CELF1, MYBPC3, SPI1, APOE, MTCH2 and RAPSN. Further functional enrichment analysis of these genes revealed the following biological pathways, including proteasome, osteoclast differentiation, hypertrophic cardiomyopathy, dilated cardiomyopathy, Epstein-Barr virus and TLV-I infection, as well as several cancer associated pathways, to be common among AD and obesity. The findings deepened our understanding on the genetic basis linking obesity and AD and may help shape possible prevention and treatment strategies.

## INTRODUCTION

Alzheimer's disease (AD) is a progressive and irreversible neurodegenerative disease discovered by Alois Alzheimer in 1906. AD is the most frequent cause of dementia and is estimated to affect 30 million subjects worldwide , and the prevalence of AD is expected to rise steadily with the aging of population in the following decades [1, 2]. Obesity is one of the most prevalent nutritional disorder around the world and the global pandemic of obesity has been a significant public health issue [3]. Obesity have been reported to act as an important risk factor for a variety of diseases, including diabetes, cardiovascular disease, hypertension, cancers, and *etc*.. [4–8]. In recent years, a growing body of evidence supports that obesity can increase the risk of developing AD [9–12]. For instance, an 18-year follow-up study found that overweight at high ages can increase the risk of developing AD in women [10]. Another study revealed that compared to the subjects with a normal body mass index (BMI), those obese (BMI > or = 30) at midlife exhibited a 3.1 fold increase in the incidence of AD, suggesting that midlife obesity can strongly predict the risk of AD [11].

As there is no effective agents to combat AD so far, more efforts have been devoted to identifying the modifiable risk factors and elucidating the underlying mechanisms to help prevent this disease. Thus, it is interesting to explore the genetic basis underlying the AD-obesity link. In the past decade, genome-wide association studies (GWAS) were successful to identify genetic variants underlying susceptibility to individual disease conditions like AD and obesity, while limited information exists on shared genetic factors between them. Therefore, in this study, we executed a bioinformatics analysis on the overlapped single nucleotide polymorphisms (SNPs), genes and biological pathways on the basis of meta-GWAS data to explore the genetic link between AD and obesity.

## RESULTS

Based on independent the International Genomics of Alzheimer's Project (IGAP) or Genetic Investigation of Anthropometric Traits (GIANT) data, we identified SNPs associated with AD or obesity with multiple p-values as cutoff criteria (Table 1). When setting at p-value of 1.0E-05 as cutoff criteria, the number of identified SNPs for the two diseases was comparable (2746 SNPs associated with AD and 4963 SNPs with obesity). Through overlapping the two sets of data, 31 SNPs were identified to be linked with both diseases, which included rs12292911, rs12798346, rs2053979, rs2293579, rs2293580, rs2856650, rs2868459, rs1057233, rs3781627, rs405509, rs4752845, rs4752856, rs4752857, rs4752990, rs4752993, rs4752994, rs4803770, rs6485758, rs10769258, rs10769262, rs10838698, rs10838699, rs7103648, rs7105851, rs755553, rs755554, rs7940536, rs874896, rs896816, rs896817, and rs11039212. When setting the threshold at p-value ≤ 1.0E-06, there was only 1 overlapped SNPs, rs405509, between AD and obesity. When setting the threshold at p-value ≤ 1.0E-07, no overlapped SNPs was found.

**Table 1:** Overlapped SNPs associated with AD and obesity with multiple cutoff *p*-values criteria

*p*-value	Number of SNPs		Overlapped SNPs
	AD	Obesity	
1.0E-7	1176	2195	0
1.0E-6	1548	3071	rs405509
1.0E-5	2746	4963	rs12292911, rs12798346, rs2053979, rs2293579, rs2293580, rs2856650, rs2868459, rs1057233, rs3781627, rs405509, rs4752845, rs4752856, rs4752857, rs4752990, rs4752993, rs4752994, rs4803770, rs6485758, rs10769258, rs10769262, rs10838698, rs10838699, rs7103648, rs7105851, rs755553, rs755554, rs7940536, rs874896, rs896816, rs896817, rs11039212.

Among the overlapped 31 SNPs, there were 28 SNPs with annotated genes. As shown in Table 2, 7 identified genes that were likely to be shared between AD and obesity. These genes included PSMC3, CELF1, MYBPC3, SPI1, APOE, MTCH2 and RAPSN. The annotated gene for the overlapped SNPs, rs405509 at p-value ≤ 1.0E-07 is APOE.

**Table 2:** Genes and biological pathways likely to be shared between AD and obesity

Biological pathways	Total pathway size	Enriched genes	Benjamini-corrected *p*-value
Proteasome	4	PSMC3	2.64E-06
Epstein-Barr virus infection	18	PSMC3, SPI1	3.05E-06
Hypertrophic cardiomyopathy	1	MYBPC3	2.04E-06
Dilated cardiomyopathy	1	MYBPC3	2.04E-06
Osteoclast differentiation	14	SPI1	3.17E-06
HTLV-I infection	14	SPI1	3.17E-06
Pathways in cancer	14	SPI1	3.17E-06
Transcriptional misregulation in cancer	14	SPI1	3.17E-06
Acute myeloid leukemia	14	SPI1	3.17E-06
Alzheimer's disease	1	APOE	1.33E-07

The functional enrichment analysis of these genes was performed using DAVID for KEGG pathway and GO term enrichment analysis. We obtained significant enrichment of the annotated genes in 10 KEGG pathways. Selected enriched KEGG pathways listed in Table 2 pertained to proteasome, osteoclast differentiation, hypertrophic cardiomyopathy, dilated cardiomyopathy, Epstein-Barr virus and TLV-I infection, and several cancer associated pathways.

## DISCUSSION

The present bioinformatics analysis explored the overlapped genes and biological pathways linking obesity and AD based on the available meta-GWAS statistics. When setting the threshold at p-value ≤ 1.0E-05, 31 overlapped SNPs were identified to be associated with both AD and obesity. These overlapped SNPs were linked to 7 genes, including PSMC3, CELF1, MYBPC3, SPI1, APOE, MTCH2 and RAPSN. Further functional enrichment analysis observed 10 biological pathways, including proteasome, osteoclast differentiation, hypertrophic cardiomyopathy, dilated cardiomyopathy, Epstein-Barr virus and TLV-I infection, as well as several cancer associated pathways.

The results were consistent with current available evidence. APOE gene encodes a protein that transports cholesterol and other types of lipids in the bloodstream. The APOE4 allele is proven to be a strong genetic risk factor for AD [13–15]. APOE gene is found to play a key role in modulating lipid metabolism and obesity [16, 17]. Interestingly, a recent study investigated the effect of interaction of APOE ε4 allele status with BMI on cognitive decline and showed that cognitive decline was differentially associated with BMI and APOE ε4 allele status [18]. Obesity is characteristic of chronic low-grade inflammation, and inflammation is an important factor in the pathogenesis of AD [19–22]. Proteasome is a multicatalytic complex to functions in the degradation of polyubiquitinated proteins. Many studies support the possible involvement of the proteasome in AD neuropathology [23–25]. The association of polymorphisms of proteasomal genes with obesity has been investigated [26,27]. In addition, increasing evidence support that osteoclast was associated with both obesity and AD [28, 29].

There are some limitations to be noted. It is worth mentioning that there are gender differences for both obesity and AD [30–32], which may arise in part from genetic factors, while the gender subgroup analysis cannot be performed. In addition, the present analysis was based on comprehensive large-scale GIANT and IGAP statistics, while other GWAS studies not included may potentially affect the outcome.

## MATERIALS AND METHODS

The data source and analytical methods were detailed as follows. First, SNPs of obesity were collected from the meta-GWAS statistics of the GIANT data set covering 322,154 participants of European descent and 17,072 participants of non-European descent [33]. SNPs of AD were obtained from the meta-GWAS statistics of the IGAP data set, comprised of 17,008 AD cases and 37,154 controls [34]. Second, through comparing the GIANT and IGAP data, the overlapped SNPs for AD and obesity were identified with multiple p-value as cutoff criteria. Third, using the database of Single-Nucleotide Polymorphism at the National Center for Biotechnology Information, the location and mapped genes of the identified overlapped SNPs for AD and obesity were analyzed. Fourth, to better understand the functions of these genes, we performed R. Kyoto Encyclopedia of Genes and Genomes (KEGG) [35] pathway and gene ontology (GO) [36] enrichment analyses using the web-based search engine Visualization and Integrated Discovery (DAVID) [37] and p-value of 0.05 was set as the threshold of significance.

## CONCLUSIONS

To summarize, by bioinformatics analysis of two meta-GWAS statistic of AD and obesity, we obtained the genetic factors that links obesity to AD. The findings have important implications for future genetic and clinical studies.
